# Prognostic value of lactate dehydrogenase for in-hospital mortality in severe and critically ill patients with COVID-19

**DOI:** 10.7150/ijms.47604

**Published:** 2020-08-19

**Authors:** Xingtong Dong, Lu Sun, Yan Li

**Affiliations:** 1 Department of Nephrology, Xuanwu Hospital, Capital Medical University, Changchun Street #45, Xicheng District 100053, Beijing, China.; 2 Department of General Disease, West Campus, Union Hospital Tongji Medical College Huazhong University of Science and Technology, China.; 3 Department of Pulmonary and Critical Care Medicine, Xuanwu Hospital, Capital Medical University, Changchun Street #45, Xicheng District 100053, Beijing, China.

**Keywords:** COVID-19, lactate dehydrogenase, prognostic value, multiple organ dysfunction

## Abstract

**Background:** Lactate dehydrogenase (LDH) has been proved to be a prognostic factor for the severity and poor outcomes of coronavirus disease 2019 (COVID-19). In most studies, patients with various levels of COVID-19 severity were pooled and analyzed which may prevent accurate evaluation of the relationship between LDH and disease progression and in-hospital death. In this study, we aimed to evaluate the association of LDH with in-hospital mortality in severe and critically ill patients with COVID-19.

**Methods:** This single-center retrospective study enrolled 119 patients. Survival curves were plotted using Kaplan-Meier method and compared by log-rank test. Multivariate Cox regression models were used to determine the independent risk factors for in-hospital mortality. Receiver-operator curves (ROCs) were constructed to evaluate the predictive accuracy of LDH and other prognostic biomarkers.

**Results:** Compared to the survival group, LDH levels in the dead group were significantly higher [559.5 (172, 7575) U/L vs 228 (117, 490) U/L, (*P* < 0.001)]. In Multivariate Cox regression, it remained an independent risk factor for in-hospital mortality (Hazard ratio 5.985, 95.0%CI: 1.498-23.905; *P*=0.011). A cutoff value of 353.5 U/L predicted the in-hospital mortality with a sensitivity of 94.4% and a specificity of 89.2% respectively.

**Conclusion:** LDH is a favorable prognostic biomarker with high accuracy for predicting in-hospital mortality in severe and critically ill patients with COVID-19. This may direct physicians worldwide to effectively prioritize resources for patients at high risk of death and to implement more aggressive treatments at an earlier phase to save patients' lives.

## Introduction

As of August 7, 2020, over nineteen million people has been confirmed infected by severe acute respiratory syndrome coronavirus 2 (SARS-CoV-2) globally and the pandemic has caused over seven hundred thousand deaths worldwide so far according to Johns Hopkins University Coronavirus Resource Center 1. To effectively prioritize resources for the patients at high risk of mortality, identification of clinical and laboratory predictors of progression towards fatal forms is urgently needed.

Lactate dehydrogenase (LDH) is a cytoplasmic enzyme which is widely expressed in tissues. The enzyme converts pyruvate, which is the final product of glycolysis, to lactate when oxygen is in short supply 2. LDH comprises two separately enclosed subunits, resulting in five isozymes. Each isozyme is expressed in a specific organ: LDH 1 in cardiomyocytes, LDH 3 in lung tissue and LDH 5 in hepatocytes 3. Increased LDH was observed in different conditions such as tissue injury, necrosis, hypoxia, hemolysis or malignancies 4, 5. Additionally, Tao *et al.* found that LDH was associated with death in patients with community acquired pneumonia (CAP) caused by viruses 6. Furthermore, LDH has been proved to be a prognostic factor with high accuracy in diseases involving multiple organ injuries such as acute heart failure (AHF) and severe acute pancreatitis (AP) 7, 8.

Coronavirus disease 2019 (COVID-19) is a disease that could cause multiple organ injuries including heart 9-12, liver and kidney injuries 13-16. Similarly, a growing number of studies demonstrated that elevated LDH value was associated with significantly increased mortality in patients with COVID-19 12, 17. But in most studies, patients with various levels of COVID-19 severity were pooled and analyzed, this may prevent accurate evaluation of the relationship between LDH and disease progression and in-hospital death because the majority of deaths were from severe and critically ill patients.

Therefore, in this study, we aimed to investigate the association of LDH with the in-hospital mortality in severe and critically ill patients with COVID-19 and estimate the predictive accuracy of LDH.

## Material and Methods

### Patients

This single-center retrospective study enrolled consecutive adult patients who were admitted to the designated hospital in Wuhan, China, with laboratory-confirmed COVID-19 and were diagnosed as severe and critically ill according to Chinese Clinical Guidance for COVID-19 Pneumonia Diagnosis and Treatment between January 29, 2020 and March 5, 2020. As it said in the guidance, severe type for adults should meet any of the following criteria: (1) shortness of breath, RR≥30 times/min (2) oxygen saturations 93% at rest (3) alveolar oxygen partial pressure/fraction of inspiration O_2_ (PaO_2_/FiO_2_) ≤300 mmHg (1 mmHg = 0.133 kPa); patients whose pulmonary imaging showed significant progression of lesion >50% within 24-48 hours. For the critically ill type, patients should meet any of the following conditions: (1) respiratory failure requiring mechanical ventilation (2) shock (3) patients combined with other organ failure needed ICU monitoring and treatment.

The patients with primary liver disease, acute myocardial infarction, decompensate heart failure, chronic kidney disease or those with missing demographic or laboratory data were excluded.

### Data collection

Demographic and clinical data including age, gender and the length of stay in hospital were collected. The baseline laboratory data on admission including hemoglobin, leukocyte count, lymphocyte count, platelet count, C-reactive protein (CRP), aspartate aminotransferase (AST), alanine aminotransferase (ALT), lactate dehydrogenase (LDH), creatine kinase (CK), Creatinine kinase-myocardial band (CK-MB) activity, α-Hydroxybutyrate (α-HBDH), albumin, serum creatinine, serum uric acid, potassium and sodium were collected at baseline. All the data were independently reviewed and entered into the computer database by two analysts (X.D. and Y.L.). Patients were divided into two groups according to the clinical outcomes of survival (discharge) or death.

### Statistical analysis

Continuous normally distributed variables and continuous non-normally distributed variables were expressed as means ± standard deviation (S.D.) and medians with range respectively. Categorical variables were presented as percentages. For normally distributed data, we used Student's *t*-test to compare the differences between the two groups. While Mann-Whitney U test was employed for non-normal data, and Chi-square test was for the categorical data. To investigate the association of LDH with in-hospital mortality in patients with COVID-19 and estimate the cumulative survival rates, survival curves were plotted using Kaplan-Meier method and compared by log-rank test. Patients who died or discharged prior to observation time were defined as censored. Multivariate Cox regression models were used to determine the independent risk factors for the in-hospital mortality. Receiver-operator curves (ROCs) were constructed to evaluate the predictive sensitivities and specificities of LDH and other factors, and the areas under the curves (AUCs) were computed. Data were analyzed using SPSS version 23.0 (IBM) and Med Calc version 19.1, all analyses were two-tailed, and a *P* value <0.05 was defined as the threshold of statistical significance.

## Results

### Baseline characteristics of patients

This study consisted of 119 adults who were severe and critically ill and 54 of them died while 65 of them survived. The causes of deaths included acute respiratory distress syndrome (ARDS), respiratory failure, shock and multiple organ dysfunction (MODS). The baseline demographic and clinical characteristics are shown in **Table 1.** There were no significant differences in hemoglobin, serum uric acid, potassium and sodium between the two groups. Compared with patients in the survival group, patients in the dead group were older [70.2±10.2 years vs 54.2±14.9 years, (*P*<0.001)], and had higher proportion of men (70.4% vs 46.2%, *P*=0.008) and longer hospital stay. In terms of laboratory findings, patients in the dead group had higher levels of leukocytes, CRP, AST, ALT, CK, CK-MB activity, α-HBDH and serum creatinine, but lower levels of lymphocytes, platelets and albumin. Notably, in the dead group, LDH level was significantly higher than that in the survival group [559.5 (172, 7575) U/L vs 228 (117, 490) U/L, *P*<0.001].

### Comparison of survival rates between groups with different levels of LDH (Kaplan-Meier survival analysis)

**Figure 1:**
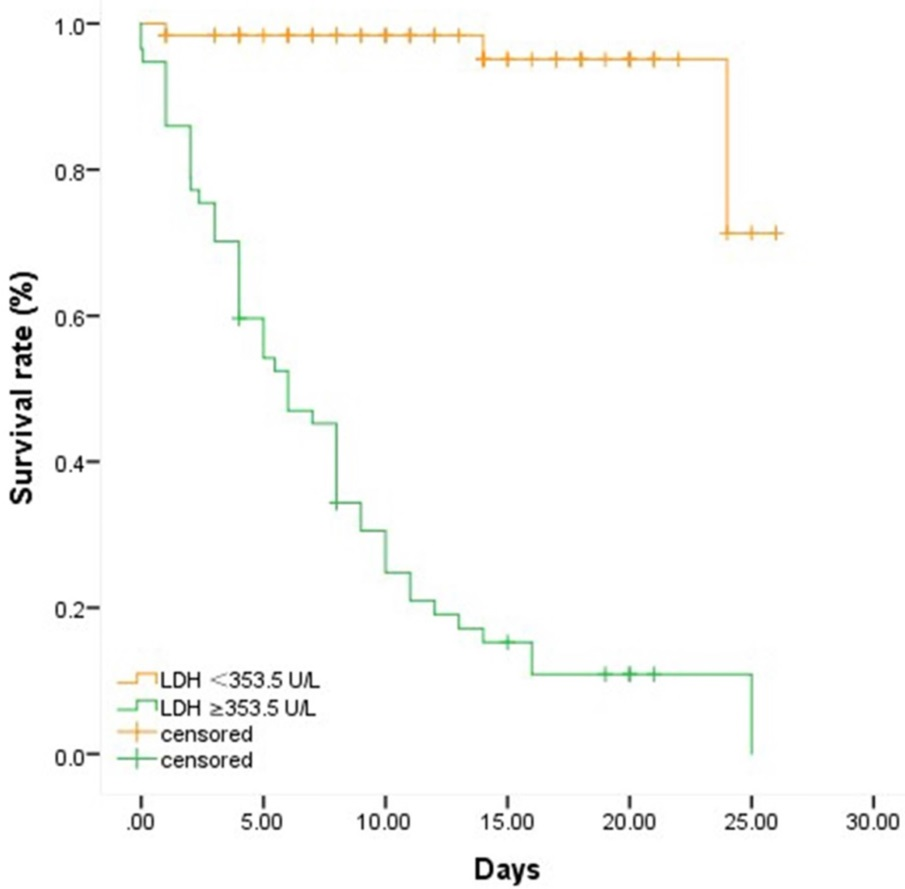
** Comparison of survival rates between groups with different levels of LDH.** Fifty-seven patients were with LDH≥ 353.5 U/L while sixty-two patients were with LDH < 353.5 U/L. We chose 26 days as the observation time point according to the longest follow-up time within our study. Patients who died or discharged prior to the observation time were defined as censored. Patients with higher level of LDH were associated with higher in-hospital mortality.

To further clarify the association of LDH with the in-hospital mortality in severe and critically ill patients with COVID-19, a cutoff value of LDH (353.5 U/L) was found using Yoden Index and Kaplan-Meier survival analysis and Log-rank test were applied. We chose 26 days as the observation time point according to the longest follow-up time within our study and divided the overall patients into two groups by the cutoff value of LDH 353.5 U/L. As depicted in **Table 2 and Figure 1**, the Kaplan-Meier survival curves showed that the survival rate in the group with LDH ≥353.5 U/L was significantly lower than that in the group with LDH <353.5 U/L (*P*<0.001).

### Comparison of clinical characteristics in patients with different LDH levels

To further investigate the clinical characteristics in patients with different LDH levels, we compared the baseline demographic and laboratory data in patients with LDH ≥353.5 U/L and patients with LDH <353.5 U/L. As demonstrated in **Table 3**, patients with LDH ≥353.5 U/L were older (67.1±11.7 years vs 56.4±16.3 years, *P*<0.001 and had higher proportion of male (71.9% vs 43.5%, *P*=0.002). They also had higher levels of leukocytes, CRP, AST, ALT, CK, α-HBDH, CK-MB activity and serum creatinine and lower levels of lymphocytes, platelets and albumin compared to patients with lower LDH level.

### Determination of independent risk factors for the in-hospital mortality by Cox regression analysis

We selected the variables which showed statistical significance (*P*<0.05) in **Table 1** and employed Cox regression analysis to find the independent risk factors for the in-hospital mortality in severe and critical ill patients with COVID-19. **Table 4** showed that categorical LDH was an independent risk factor of the in-hospital mortality. The risk of death in patients with LDH ≥353.5 U/L was as 5.985 times as patients with LDH <353.5 U/L. Besides, AST, ALT and albumin were also independent risk factors for the in-hospital mortality in severe and critically ill patients with COVID-19.

### Predictive accuracies of different biomarkers for the in-hospital mortality in sever and critically ill patients with COVID-19

**Figure 2:**
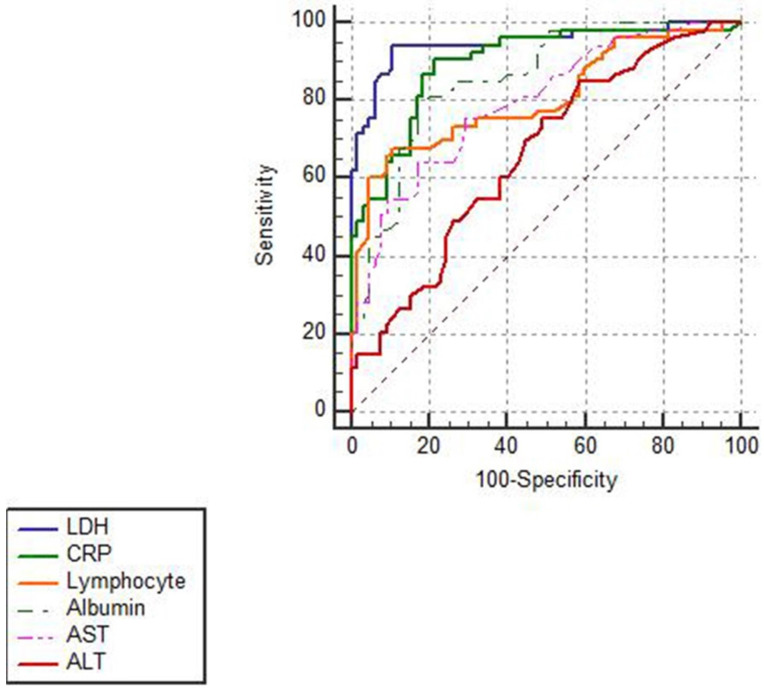
** ROC analysis of different biomarkers in predicting in-hospital mortality in patients with severe and critically ill COVID-19.** The ROCs of different biomarkers showed that LDH had the biggest AUC and a credible prognostic value with a high sensitivity and specificity for predicting in-hospital mortality in patients with severe and critically ill COVID-19.

In **Figure 2**, ROCs were obtained from the risk factors which were statistically significant in Cox regression analysis and from the well-established early warning indicators CRP and lymphocyte that recommended in Chinese Clinical Guidance for COVID-19 Pneumonia Diagnosis and Treatment (7th edition). As shown in **Table 5**, LDH showed the biggest AUC of 0.949, with a sensitivity of 94.9% and a specificity of 89.2% and the optimal threshold was 353.5 U/L. Notably, LDH had a higher prognostic value than lymphocyte (*P*<0.05) and the similar value with CRP (*P*>0.05) for predicting the in-hospital mortality in severe and critically ill patients.

## Discussion

The in-hospital mortality of severe and critically ill patients with COVID-19 could be up to almost 40% 18, the mortality in our study was even higher (45%). Many studies during the early pandemic reported the elevated LDH in severe or deceased cases 12, 19, 20, especially in cases involving cardiac injury 20-23. As the increasing experience with COVID-19 worldwide, numerous studies found that LDH was associated with the severity and poor outcomes of COVID-19. Shi *et al*. 24 demonstrated that high LDH level was an independent risk factor for the exacerbation in mild COVID-19 patients. Poggiali* et al.* 25 reported that LDH may be related to respiratory function (PaO_2_/FiO_2_) and be a predictor of respiratory failure in COVID-19 patients. Han *et al*. 26 argued that LDH could be identified as a powerful predictive factor for early recognition of lung injury and severe COVID-19 cases. However, there have been few studies focusing on the specific patient population where the most deaths came from. In the present study, we determined the association of LDH with the in-hospital mortality in severe or critically patients with COVID-19 and demonstrated that LDH level was an independent risk factor of in-hospital mortality. Furthermore, we found a cutoff value of LDH to predict the in-hospital mortality in severe or critically patients with COVID-19. Physicians need to implement more aggressive treatments to patients with LDH ≥353.5 U/L.

Additionally, we also found that various organ specific enzymes or biomarkers such as AST, ALT, CK-MB, and serum creatinie were significantly higher in the dead group which corresponded to the previous findings about the involvement of multiple organ injuries in COVID-19. Except the cardiac injuries, Zhang *et al.* reported that the incidence of hepatic abnormalities significantly increased after infection with COVID-19 13. Xu *et al.* have also reported steatosis and liver injury through the liver biopsy of a patient with COVID-19 14. Beside of liver injuries, some researchers have reported an increased incidence of acute renal injury following COVID-19, which could be attributed to the influence of SARS-CoV-2, the inflammation induced by this disease, or a synergistic effect of both on kidneys 15, 16.

CRP and lymphocyte have been confirmed as prognostic biomarkers since the outbreak of COVID-19 and they were also recommended as the early warning indicators for severe and critically ill cases by Chinese Clinical Guidance for COVID-19 Pneumonia Diagnosis and Treatment (7th edition). Han *et al.* 26 compared LDH with other prognostic biomarkers including CRP, lymphocyte and AST in predicting severe COVID-19 cases in patients with various levels of COVID-19 severity and demonstrated that LDH had higher accuracy than CRP and lymphocyte in predicting the severity. However, our study showed that LDH had a higher prognostic accuracy than lymphocyte and a similar accuracy as CRP for predicting the in-hospital mortality in severe and critically ill patients with COVID-19. Our finding may provide more accurate evaluation of the relationship between LDH and disease progression and in-hospital death.

## Limitation

This study has several limitations. First, the study was conducted at a single-center hospital with limited sample size, a large-scale and multiple center study was needed to further confirm the prognostic value of LDH. Second, selection bias might occur for this retrospective study, and further prospective studies were needed. Third, some other specific information regarding inflammation and cardiac injuries such as interleukin 6 and troponin I (TnI) were not available owing to the limited conditions in the hospital. Fourth, more detailed clinical characteristics of the patient population such as acute ischemic stroke, new confusion, inability to wake or stay awake, bluish lips or face were unavailable at the time of analysis.

## Conclusion

LDH is a favorable prognostic biomarker with high accuracy for predicting the in-hospital mortality in severe and critically ill patients with COVID-19. This easily available biomarker will direct physicians worldwide to effectively prioritize resources for patients at high risk of mortality and to implement more aggressive treatments at an earlier phase to save patients' lives especially in the poor regions.

## Figures and Tables

**Table 1:** Comparison of baseline demographic and clinical characteristics between dead patients and survived patients

	Dead (n=54)	Survival (n=65)	*P* value
Male/female (%)	70.4	46.2	0.008
Age (years)	70.2±10.2	54.2±14.9	<0.001
Length of stay (days)	5 (0,25)	14 (1,26)	<0.001
Hemoglobin (g/L)	126.4±23.0	125.8±23.4	0.893
Leukocytes (×10^9^/L)	10.0±4.5	5.0±1.9	<0.001
Lymphocytes (×10^9^/L)	0.6 (0.2, 2.0)	1.0 (0.4, 2.4)	<0.001
Platelets (×10^9^/L)	152 (29, 444)	211 (62, 478)	0.001
CRP (mg/L)	77.8 (0.1, 187.5)	11.3 (0.1, 93.1)	<0.001
AST (U/L)	52.5 (17, 2487)	28 (12, 91)	<0.001
ALT (U/L)	45 (13, 1747)	27 (9, 92)	0.002
LDH (U/L)	559.5 (172, 7575)	228 (117, 490)	<0.001
Creatine kinase (U/L)	162 (22, 1803)	63 (23, 2131)	<0.001
CK-MB activity (U/L)	20 (9, 122)	12 (5, 65)	<0.001
α-HBDH (U/L)	479.2±188.1	205.7±70.2	<0.001
Albumin (g/L)	26.6±5.1	33.0±4.2	<0.001
Serum creatinine (umol/L)	77.5 (47.3, 1040.8)	64.4 (43.2, 807.9)	<0.001
Serum uric acid (umol/L)	238.2 (92.9, 930.9)	229.2 (99.6, 535.4)	0.278
Potassium (mmol/L)	4.1±0.7	3.8±0.5	0.181
Sodium (mmol/L)	138.1±5.4	134.0±23.2	0.204

Comparison of demographic and clinical characteristics between dead patients and survived patients showed a significant difference in LDH level and other characteristics including sex, age, length of stay, leukocytes, lymphocytes, platelets, CRP, AST, ALT, CK, CK-MB activity, α-HBDH, albumin and serum creatinine.

**Table 2:** Comparison of survival rates in patients with different levels of LDH

LDH grouping	χ^2^	*P* value
≥353.5 ( U/L)	77.732	<0.001
<353.5 ( U/L)		

Patients with LDH ≥ 353.5 U/L had significantly higher mortality than those with LDH < 353.5 U/L.

**Table 3:** Comparison of clinical characteristics in patients with different levels of LDH

	LDH ≥ 353.5U/L	LDH < 353.5U/L	*P* value
Cases (n)	57	62	
Male/female (%)	71.9	43.5	0.002
Age (years)	67.1±11.7	56.4±16.3	<0.001
Hemoglobin (g/L)	129.7±19.6	122.8±25.7	0.105
Leukocytes (×10^9^/L)	9.5±4.7	5.2±2.0	<0.001
Lymphocytes (×10^9^/L)	0.6 (0.2,2.0)	1.1 (0.3, 2.4)	<0.001
Platelets (×10^9^/L)	161.0 (29.0, 450.0)	212.0 (56.0, 478.0)	0.002
CRP (mg/L)	77.6 (0.1,187.5)	11.1 (0.1, 93.1)	<0.001
AST (U/L)	54 (17.0,2487.0)	26.5 (12.0, 67.0)	<0.001
ALT (U/L)	49.0 (14.0,1747.0)	24.0 (9.0, 92.0)	<0.001
Creatine kinase (U/L)	164.5 (22.0, 2131.0)	63.0 (23.0,515.0)	<0.001
CK-MB activity (U/L)	20.0 (9.0,122.0)	12.0 (5.0,65.0)	<0.001
α-HBDH (U/L)	482.4±173.9	189.6±48.6	<0.001
Albumin (g/L)	27.4±4.8	32.7±5.1	<0.001
Serum creatinine (umol/L)	78.2 (47.3,1040.8)	63.7 (43.2, 807.9)	<0.001
Serum uric acid (umol/L)	250.2 (92.9,930.9)	227.5 (99.6, 535.4)	0.075
Potassium (mmol/L)	4.0±0.7	3.9±0.5	0.187
Sodium (mmol/L)	138.4±5.2	133.6±23.7	0.128

Patients with higher level of LDH had significantly poorer heart and liver function, higher level of CRP and lower level of lymphocyte.

**Table 4:** Multivariate Cox regression analysis of risk factors for in-hospital mortality in sever and critically ill patients with COVID-19

Variables	B	*P* value	Exp (B)	95.0%CI for Exp (B)
Male/female (%)	0.680	0.107	1.973	0.864-4.507
Age (years)	0.013	0.469	1.013	0.979-1.048
Leukocytes (×10^9^/L)	0.023	0.574	1.023	0.945-1.107
Lymphocytes (×10^9^/L)	-0.478	0.384	0.620	0.212-1.817
Platelets (×10^9^/L)	-0.002	0.405	0.998	0.995-1.002
CRP (mg/L)	0.007	0.091	1.007	0.999-1.015
AST (U/L)	0.017	0.018	1.018	1.003-1.032
ALT (U/L)	-0.020	0.035	0.980	0.962-0.999
LDH (≥353.5 U/L, <353.5U/L)	1.789	0.011	5.985	1.498-23.905
Creatine kinase (U/L)	0.001	0.095	1.001	1.000-1.002
CK-MB activity (U/L)	0.019	0.075	1.019	0.998-1.041
α-HBDH (U/L)	0.001	0.564	1.001	0.998-1.004
Albumin (g/L)	-0.168	<0.001	0.846	0.784-0.912
Serum creatinine (umol/L)	-0.001	0.544	0.999	0.998-1.001

AST, ALT, LDH and albumin were independent risk factors of in-hospital mortality in sever and critically ill patients with COVID-19.

**Table 5:** ROC analysis of different biomarkers for predicting the in-hospital mortality of severe and critically ill patients with COVID-19

	AUC (95%CI)	Cutoff	Sensitivity	Specificity
LDH ( U/L)	0.949	353.5	94.4	89.2
CRP(mg/L)	0.896	30.95	90.6	78.5
Lymphocyte (×10^9^/L)	0.805	0.7	67.9	89.2
Albumin(g/L)	0.857	29.4	79.2	83.1
AST( U/L)	0.796	43	64.8	83.1
ALT( U/L)	0.662	22	84.9	41.5

LDH was a favorable biomarker with the highest sensitivity and specificity for predicting the in-hospital mortality of severe and critically ill patients with COVID-19.
